# Noninvasive Imaging of Conjunctival Goblet Cells as a Method for Diagnosing Dry Eye Disease in an Experimental Mouse Model

**DOI:** 10.1167/tvst.12.12.22

**Published:** 2023-12-27

**Authors:** Jeongho Kim, Jungbin Lee, Seonghan Kim, Sook Hyun Yoon, Yeong Chae Jo, Ki Hean Kim, Hong Kyun Kim

**Affiliations:** 1 Bio-Medical Institute, Kyungpook National University Hospital, Jung-gu, Daegu, Republic of Korea; 2 Department of Mechanical Engineering, Pohang University of Science and Technology, Pohang, Gyeongbuk, Republic of Korea; 3 Department of Ophthalmology, Daegu Catholic University School of Medicine, Nam-gu, Daegu, Republic of Korea; 4 Department of Ophthalmology, Maryknoll Medical Center, Jung-gu, Busan, Republic of Korea; 5 Department of Ophthalmology, School of Medicine, Kyungpook National University, Jung-gu, Daegu, Republic of Korea

**Keywords:** moxifloxacin-based fluorescence microscopy (MBFM), conjunctival goblet cells (GCs), dry eye disease (DED), diagnostic imaging

## Abstract

**Purpose:**

The purpose of this study was to evaluate a noninvasive conjunctival goblet cell (GC) imaging method for assessing dry eye disease (DED) in an experimental mouse model.

**Methods:**

Moxifloxacin-based fluorescence microscopy (MBFM) was used to examine GCs noninvasively in 56 mice. Forty-two (42) DED-induced mice were divided into 2 groups and treated topically for 14 days with cyclosporine (CsA) or normal saline (NS). In vivo MBFM imaging and clinical DED evaluations were performed and goblet cell density (GCD) and goblet cell area (GCA) were obtained and compared with histological GCD using periodic acid-Schiff (PAS) staining. Correlation and receiver operating characteristic (ROC) analyses showed MBFM's high diagnostic value.

**Results:**

The GCD and GCA of the DED mice obtained from in vivo MBFM imaging were highly correlated with clinical DED parameters and GCD obtained from PAS histology. The therapeutic effect of CsA, as observed by in vivo MBFM, was significant with respect to that of NS treatment. The ROC curves derived from in vivo MBFM showed high diagnostic value in assessing DED.

**Conclusions:**

The proposed noninvasive method has high diagnostic value in assessing the severity of DED and the effect of treatment for this disease.

**Translational Relevance:**

A noninvasive imaging method using moxifloxacin-based fluorescence microscopy was evaluated for assessing DED in an experimental mouse model. The method showed high diagnostic value in assessing the severity of DED and the effect of treatment, bridging the gap between basic research and clinical treatment. The study provides a promising tool for diagnosing and monitoring DED.

## Introduction

Dry eye disease (DED) is a common multifactorial disorder of the ocular surface caused by the loss of homeostasis of the tear film and is associated with a wide variety of ocular symptoms.^1^ Any disturbances in the tear film, including lacrimal secretion deficiency, meibomian gland dysfunction (MGD), neurosensory impairment, and mucous layer alteration, can result in DED. Mucous gel plays a key role in protecting the ocular surface by maintaining the tear film and lubricating the eye lids for blinking. Conjunctival goblet cells (GCs), the main source of gel-forming mucins, are one of the major cell types in the conjunctival epithelium and are distributed individually or in clusters.^2^ In dry eye conditions, tear film hyperosmolarity induces an inflammatory response, leading to reduced expression of glycocalyx mucins, apoptotic death of the surface epithelium, and loss of GCs.^3^ The reduction in GC density has been demonstrated to be proportional to the severity of DED.^4^ Therefore, examination of the GCs could be one of the main targets for the diagnosis and treatment monitoring of DED.

Previously, we reported a high-contrast imaging method for visualizing conjunctival GCs called moxifloxacin-based fluorescence microscopy (MBFM).^5^^,^^6^ MBFM is a fluorescence imaging technique based on the GC-specific labeling of intrinsically fluorescent moxifloxacin ophthalmic solution. Three-dimensional confocal fluorescence microscopy was used for the initial verification of the efficacy of MBFM with reference to periodic acid-Schiff (PAS) histology. Axially swept wide-field fluorescence microscopy (AS-WFFM) was developed for noncontact GC imaging of live animals*.*^5^^,^^6^ MBFM provides high-contrast GC images sufficient to evaluate the status of GCs. MBFM was applied to diseased animal models, including chemical burn mouse models generated with topical sodium hydroxide^6^ and ocular surface damage rabbit models generated with topical povidone iodine ophthalmic antiseptic. In vivo longitudinal MBFM revealed the loss and recovery of conjunctival GCs in these animal models.^6^^,^^7^

In this study, we applied MBFM to dry eye-induced mouse models to test its feasibility as a method for assessing DED in terms of both diagnosis and treatment monitoring. Dry eye conditions were induced by applying scopolamine patches and housing the mice in a controlled environmental chamber (CEC) with constant air flow for 10 days. Then, the mice were treated with cyclosporin A (CsA) for 14 days. MBFM was used to assess the status of the conjunctival GCs during treatment, and various clinical methods were used to evaluate the dry eye condition. PAS histology was used for performing conventional goblet cell density (GCD) analysis during the treatment.

## Materials and Methods

### Imaging System

In vivo GC imaging of mouse models was conducted by using AS-WFFM.^6^ AS-WFFM is a type of wide-field fluorescence microscopy with an extended depth of field (DOF) of 1 mm for imaging GCs on arbitrarily tilted conjunctiva in live animal models without physical contact. The extended DOF was achieved by axially sweeping the focal plane and acquiring multiple images at different depths. The acquired images were processed to generate single all-in-focus surface images. AS-WFFM was performed with a 405 nm LED (M405L3, Thorlabs, Germany) as the excitation light source and a 10 × objective lens (PLAN 10 × 0.25 NA, Olympus, Japan) for sample illumination and emission light collection. A scientific CMOS (sCMOS) camera (PCO.edge 4.2, PCO AG, Germany) was used for the sensitive collection of emission light with wavelengths longer than 430 nm. For axial sweeping, a motorized translation stage moved the objective lens axially and stepwise in synchronization with image acquisition. The AS-WFFM device has an imaging field of view (FOV) of 1.2 mm × 1.2 mm, an image resolution of 1.2 µm, and an imaging speed of 1 fps. The typical excitation energy used in the imaging was approximately 0.3 J/cm^2^, which is approximately 60 × lower than the reported damage threshold.^8^

### Animals

This study was conducted in accordance with the ARVO Statement for the Use of Animals in Ophthalmic and Vision Research and was approved by the Institutional Animal Care and Use Committee of Daegu-Gyeongbuk Medical Innovation Foundation, Laboratory Animal Center (IACUC No. DGMIF-20030605-01)*.* Fifty-six male BALB/c mice (Orient Bio, Korea) aged 8 to 11 weeks were used in this study. The mice were acclimated for 1 week. Dry eye conditions were induced in 49 of these mice by applying a transdermal scopolamine patch (Kimite, MyoungMoon, Korea) on the tail and housing the mice in a controlled environmental chamber (relative humidity = 18.5% ± 5%; airflow rate = 20 L/min; temperature = 23°C ± 2.5°C) for 10 days*.*^9^ The scopolamine patch was replaced every 2 days during the DED induction period in the chamber.

**Figure 1.**
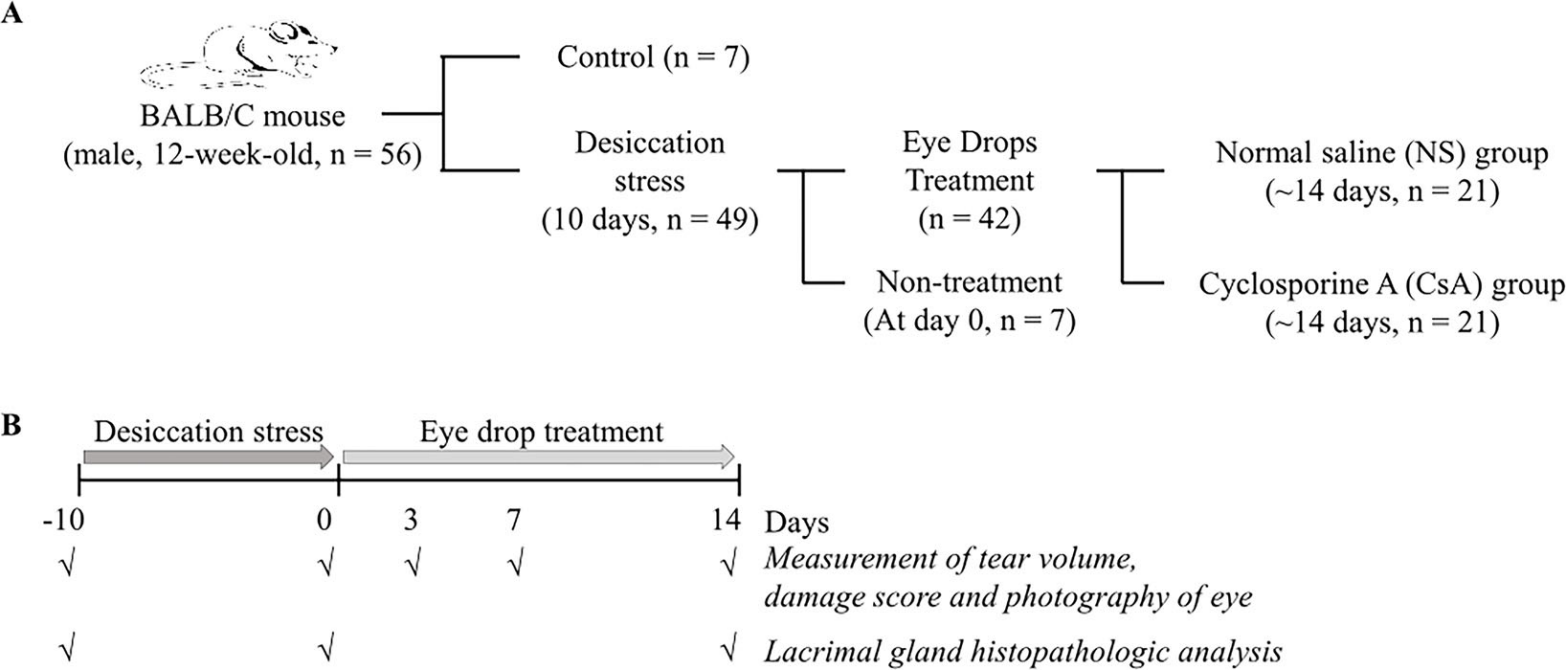
Group classification and experimental overview. A total of 56 mice were included in the study. Among them, 49 mice were induced with dry eye for 10 days. They were then divided into 2 groups: one received cyclosporine (CsA) treatment, and the other received normal saline (NS) for 3, 7, and 14 days. Clinical battery tests and conjunctival goblet cell analysis were conducted after the treatment period. Additionally, seven mice without dry eye induction served as the negative control group, whereas seven mice with induced dry eye served as the positive control group (*n* = 7).

After 10 days of desiccating stress, the DED mice were randomly assigned to 2 groups and were treated with normal saline (NS; *n* = 7, 5 µL of NS) and CsA (*n* = 7, 5 µL of CsA 0.1%, Ikervis; Santen Pharmaceutical, Japan) for 14 days. In vivo MBFM imaging and clinical DED evaluations were performed both before treatment and on days 3, 7, and 14 of the treatment periods. At each time point during the treatment, seven mice were euthanized for histological GC examination with PAS staining (Fig. 1).

### In Vivo GC Imaging Procedure

In vivo conjunctival GC imaging was performed with the AS-WFFM system. The mice were anesthetized with intraperitoneal injections of ketamine (50 mg/kg; Yuhan Corperation, Korea) and xylazine (20 mg/kg, Rompun; Careside, Korea) and positioned under the system. Subsequently, the upper eyelid was everted and fixed with a bent needle to expose both the fornix and palpebral conjunctiva for imaging. A drop of moxifloxacin ophthalmic solution (Vigamox; Alcon Laboratories, USA) was topically instilled; after 90 seconds, any remaining moxifloxacin solution was removed with 3 washes with NS. In vivo GC images were captured at 1 fps, and the GC images were analyzed by using the ImageJ program (National Institutes of Health [NIH]).^10^ The GCD and GC area (GCA) were calculated in a predefined 450 µm × 450 µm area in the central upper palpebral conjunctiva. The GCD was calculated as the number of GC clusters, and the GCA was calculated as the area of GC clusters. In order to minimize potential biases and ensure the validity of the results, a single blind strategy was implemented in this animal experiment. Researchers who are involved in the evaluation of GC imaging were unaware of the group assignments throughout the study. The experimental and control groups were coded using anonymized labels, and the codes were only revealed after data collection and analysis were completed.

### Measurement of Clinical DED Parameters

For comparison with in vivo MBFM, various clinical DED evaluation metrics, including the degree of corneal irregularity, tear production rate, and corneal staining score, were collected during the treatment period. The degree of corneal irregularity was used to measure tear film instability. A slit lamp microscope was used to capture an image of white ring light reflected on the cornea of the mouse immediately after anesthesia. Corneal irregularity was evaluated as the average number of distorted quarters in the reflective ring image on a 5-point scale, as previously described.^11^ The tear production rate was analyzed using phenol red threads (PRT; Zone-Quick; Oasis, USA).^12^ After lifting the lower eyelid of the mouse, a thread was hung on the lateral canal, and tears were absorbed for 1 minute. The length of the thread that was discolored to red by the absorbed tears was measured and quantified. The corneal staining scores were calculated after the tear-volume measurement.^13^ After dissolving a lissamine green ophthalmic strap (Optitech Eyecare, India) in 2 to 3 drops of phosphate buffered saline (PBS), 5 µL of lissamine green solution was instilled into the mouse’s conjunctival sac. The entire cornea was stained for 30 seconds by carefully blinking 3 times, and the remaining lissamine green solution was removed using a cotton swab. The stained corneal surface was photographed using a slit-lamp microscope. The photographs were analyzed to evaluate corneal damage by using the Expanded National Eye Institute (NEI)/Industry Workshop corneal fluorescein staining (CFS) scale.^14^ The scoring of clinical DED parameters was performed by two ophthalmologists who were unaware of the experimental groups regarding DED induction and treatment.

### Histology

Both before and during the treatment period, mice were euthanized for histological analysis. Tissue specimens, including the eyeball, conjunctiva, and eyelid, were collected and fixed in Davison's fixative solution. After 24 hours, the fixative solution was changed to 10% neutral buffered formalin. The eyeball specimens were trimmed to remove the retina, embedded in paraffin, and sectioned. Thin, 6 µm sections were stained with PAS and imaged under wide-field microscopy (Nikon Ti-S inverted, Japan).

### Statistical Analysis

All data are expressed as the mean ± SD and were analyzed using GraphPad Prism software (Prism for Windows 10, version 8.0; GraphPad, USA) for Microsoft Windows 10. Differences between the groups were analyzed using the unpaired Student's *t*-test or multiple t test. Statistical significance was determined using the Mann–Whitney *U* test and the Holm–Sidak method and was defined as *P* < 0.05. Spearman's rank correlation coefficient was calculated to determine the correlation between the results of in vivo MBFM imaging and the other DED evaluation experiments. The ability of MBFM GC imaging to diagnose DED was assessed with receiver operating characteristic (ROC) curve analysis using SPSS software (IBM SPSS Statistics for Windows, version 26.0.; IBM Corp., Armonk, NY, USA). NEI/CFS scores exceeding 4 were defined as a standard diagnosis of DED, and the specificity, sensitivity, and cutoff scores for each DED parameter were calculated. Statistical significance was defined as *P* < 0.05.

## Results

### In Vivo GCD and GCA Analysis of DED Mice During Treatment

**Figure 2.**
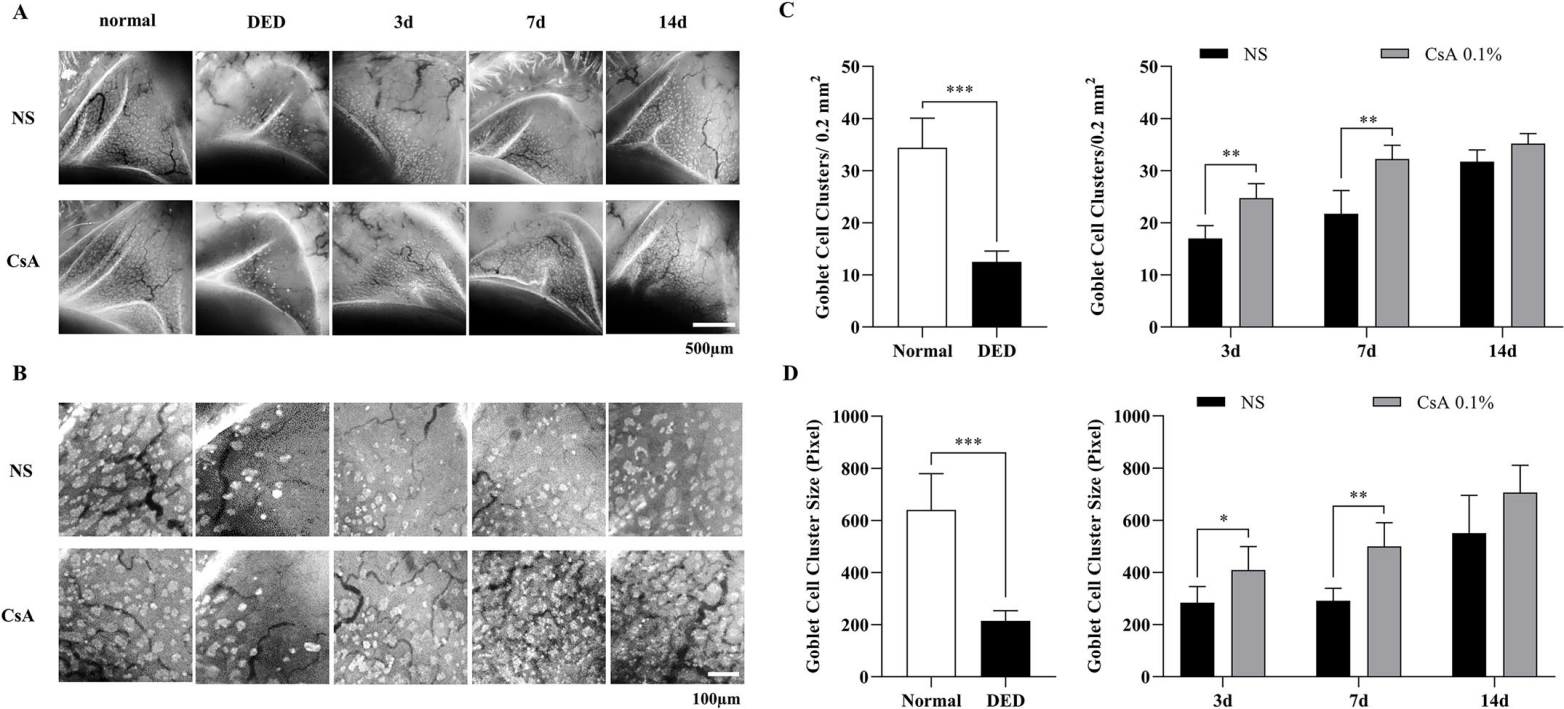
In vivo conjunctival goblet cell (GC) images and quantitative analysis results of DED mice after DED induction and during treatment. (**A**) Representative conjunctival GC images in all experimental groups, including normal mice, DED mice treated with NS, and DED mice treated with CsA after DED induction and on days 3, 7, and 14 of the treatment period. (**B**) Magnified GC images from the selected region of (**A**) for the calculation of GC density (GCD) and area (GCA). (**C**) Conjunctival GCDs of the experimental groups from the magnified images in (**B**). (**D**) Conjunctival GCAs of the experimental groups from the magnified images in (**B**). Data are presented as the mean ± standard deviation, and data between normal and DED groups were analyzed by the *t*-test and Mann–Whitney *U* test. Data on days 3, 7, and 14 of treatment between the NS and CsA groups were analyzed by the multiple *t* test. Statistical significance was assessed using the Holm–Sidak method, *N* = 7, *P < 0.05.

MBFM was used to noninvasively assess conjunctival GCs in normal and DED-induced mice. The DED mice were divided into 2 groups treated with either NS or CsA for 2 weeks. Both before and during the treatment, in vivo MBFM imaging was conducted longitudinally, and the GCD and GCA were calculated from the MBFM images. The results of in vivo MBFM imaging and analysis, presented in Figure 2, indicate that DED-induced mice presented with a much smaller number of GCs and significantly lower GCD (*P* < 0.0001) and GCA values (*P* < 0.0001) than the normal mice. The GCD values of the normal and DED-induced mice were 34.4 ± 5.7 and 12.5 ± 2.1, respectively, whereas the corresponding GCA values were 640 ± 140 and 215 ± 39, respectively.

During the treatment period, in vivo MBFM images showed that both treatment groups presented with GC recovery, although at different rates. The CsA-treated DED group showed significantly higher GCD and GCA values than the NS-treated DED group on all imaging days during the treatment period. The GCD values of the NS-treated DED group were 17.0 ± 2.5, 21.8 ± 4.4, and 31.8 ± 2.2 on days 3, 7, and 14 of treatment, respectively, whereas those of the CsA-treated DED group were 24.8 ± 2.8, 32.3 ± 2.6, and 35.2 ± 1.9. The GCD value of the CsA-treated DED mice on day 14 was similar to that of normal mice, indicating complete recovery. The GCD values of the 2 treatment groups were significantly different on all imaging days, with *P* values < 0.0057, < 0.0065, and < 0.057 on days 3, 7, and 14, respectively. The GCA values of the NS-treated DED group were 285 ± 62, 291 ± 48, and 555 ± 140 on days 3, 7, and 14 of the treatment period, respectively, whereas those of the CsA-treated DED group were 410 ± 89, 500 ± 91, and 707 ± 104 on days 3, 7, and 14, respectively. The GCA value of the CsA-treated DED mice on day 14 was within the level of normal mice, indicating complete recovery. The GCA values of the 2 groups were significantly different on days 3, 7, and 14: *P* < 0.026, P < 0.0013, and *P* < 0.068, respectively. Both the NS-treated and CsA-treated DED groups had fully recovered on day 14, with no statistically significant difference between the 2 groups.

### Conventional Ex Vivo GCD Analysis of DED Mice During Treatment

**Figure 3.**
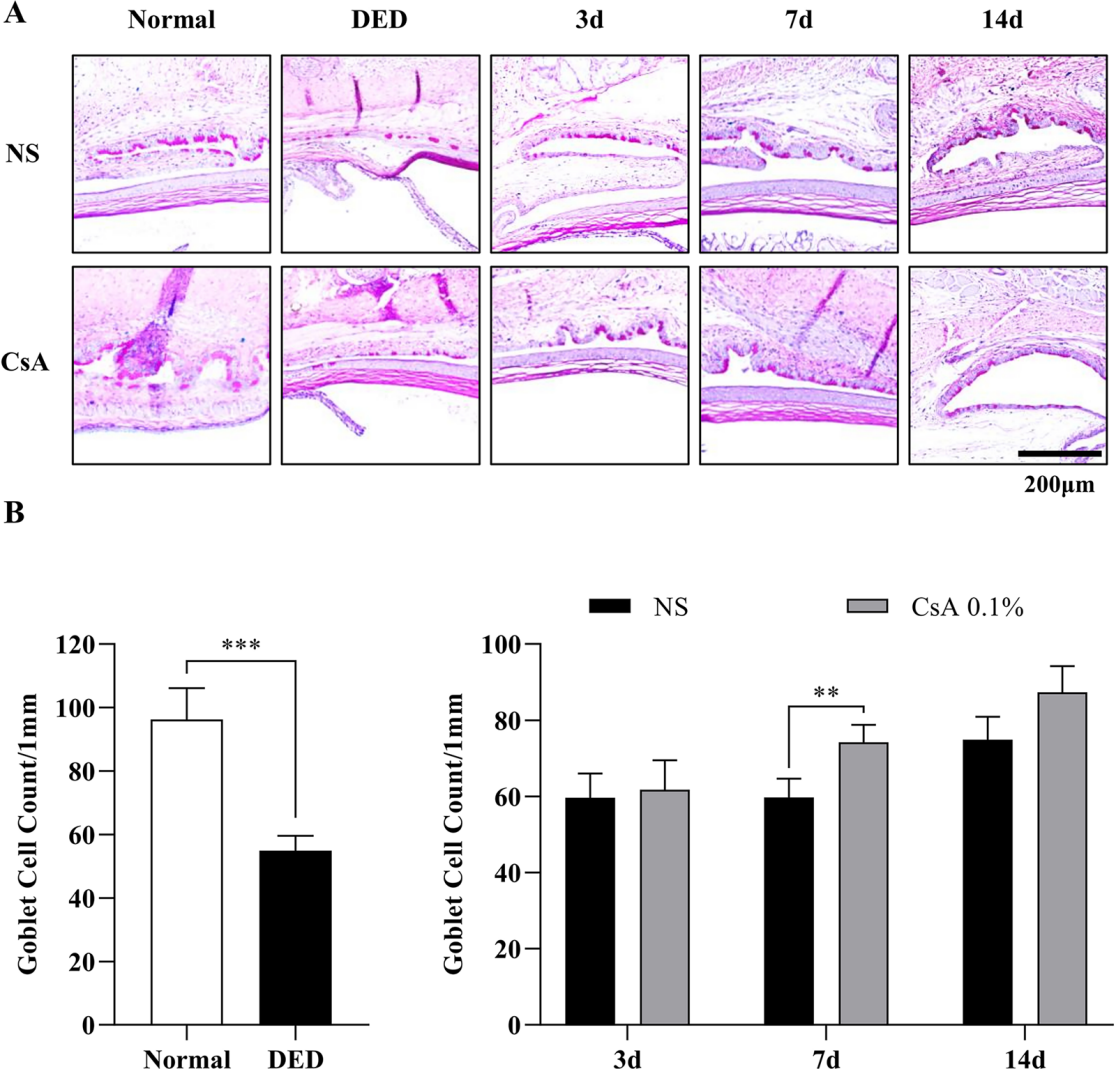
Histological analysis of goblet cells (GCs) and their density in DED mice after induction and during treatment. (**A**) Periodic acid-Schiff (PAS)-stained histological images in all the experimental groups of normal mice, NS-treated DED mice, and CsA-treated DED mice. (**B**) GC densities between the normal and DED groups and longitudinal changes in GC densities in the NS-treated and CsA-treated DED groups. GC density was obtained by counting PAS-positive GCs divided by the epithelium length. Data are presented as the mean ± standard deviation, and GC densities of the normal and DED groups were analyzed by the *t* test and Mann–Whitney *U* test. The GC densities of the NS-treated and CsA-treated DED groups on days 3, 7, and 14 of the treatment period were analyzed by performing the multiple *t* test. Statistical significance was determined using the Holm–Sidak method, *N* = 7, *P < 0.05.

To verify the in vivo MBFM imaging and GC analysis results, conventional GCD analysis was performed through histology with PAS staining (Fig. 3A). The histology-derived GCD (hGCD) of the DED-induced mice was 54.9 ± 4.8, approximately half that of the normal mice (96.3 ± 9.9). The hGCD values between the two groups were significantly different (*P* < 0.0001; Fig. 3B). On day 3 of the treatment period, the hGCD values of the NS-treated DED group and the CsA-treated DED group were 59.6 ± 6.4 and 61.8 ± 7.7, respectively. On days 7 and 14, the hGCD values of the NS-treated DED group increased to 59.7 ± 5.0 and 74.9 ± 6.0, respectively, and those of the CsA-treated DED group increased to 74.2 ± 4.6 and 87.3 ± 6.7, respectively. The hGCD values of the 2 treatment groups were significantly different on the latter 2 days (day 7, *p* < 0.0027 and day 14, *P* < 0.016). The hGCD of the CsA-treated DED group increased faster than that of the NS-treated group, and these changes in hGCD were consistent with the GCD and GCA results from in vivo MBFM.

### Analysis of Clinical DED Evaluation Parameters of DED Mice During Treatment: Corneal Irregularity Score, Corneal Staining Score, and PRT

**Figure 4.**
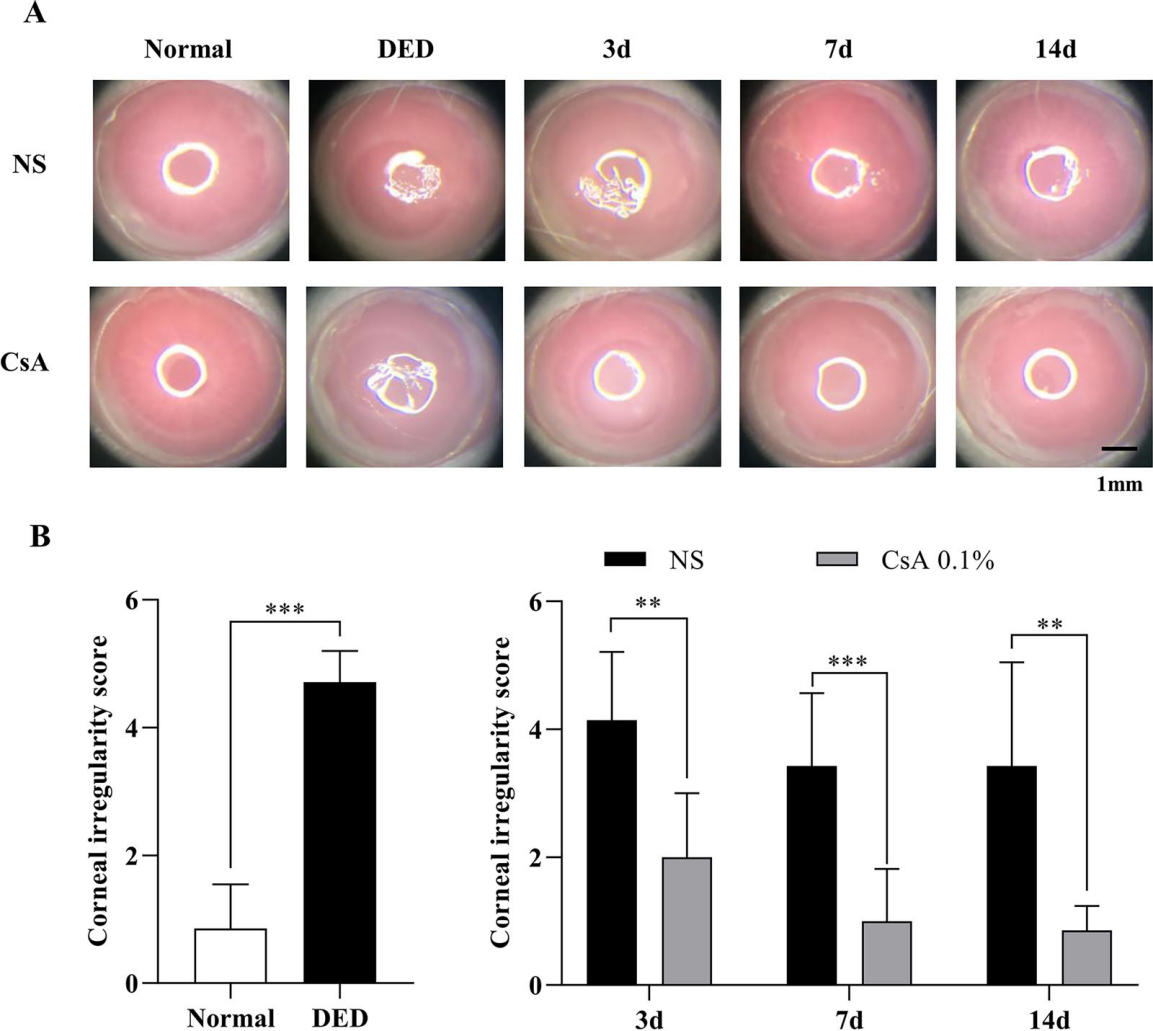
Corneal irregularity analysis in DED mice after DED induction and during treatment. (**A**) Ring-light reflection images of the corneas of normal and DED mice immediately after DED induction and on days 3, 7, and 14 of treatment with NS and CsA. (**B**) Corneal irregularity scores between normal and DED mice and the longitudinal changes in irregularity scores in the NS-treated and CsA-treated DED groups. Data are presented as the mean ± standard deviation, and normal and DED groups were compared with the *t* test and Mann–Whitney *U* test. Longitudinal data on days 3, 7, and 14 were compared with the multiple *t* test. Statistical significance was assessed using the Holm–Sidak method, *N* = 7, *P < 0.05.

**Figure 5.**
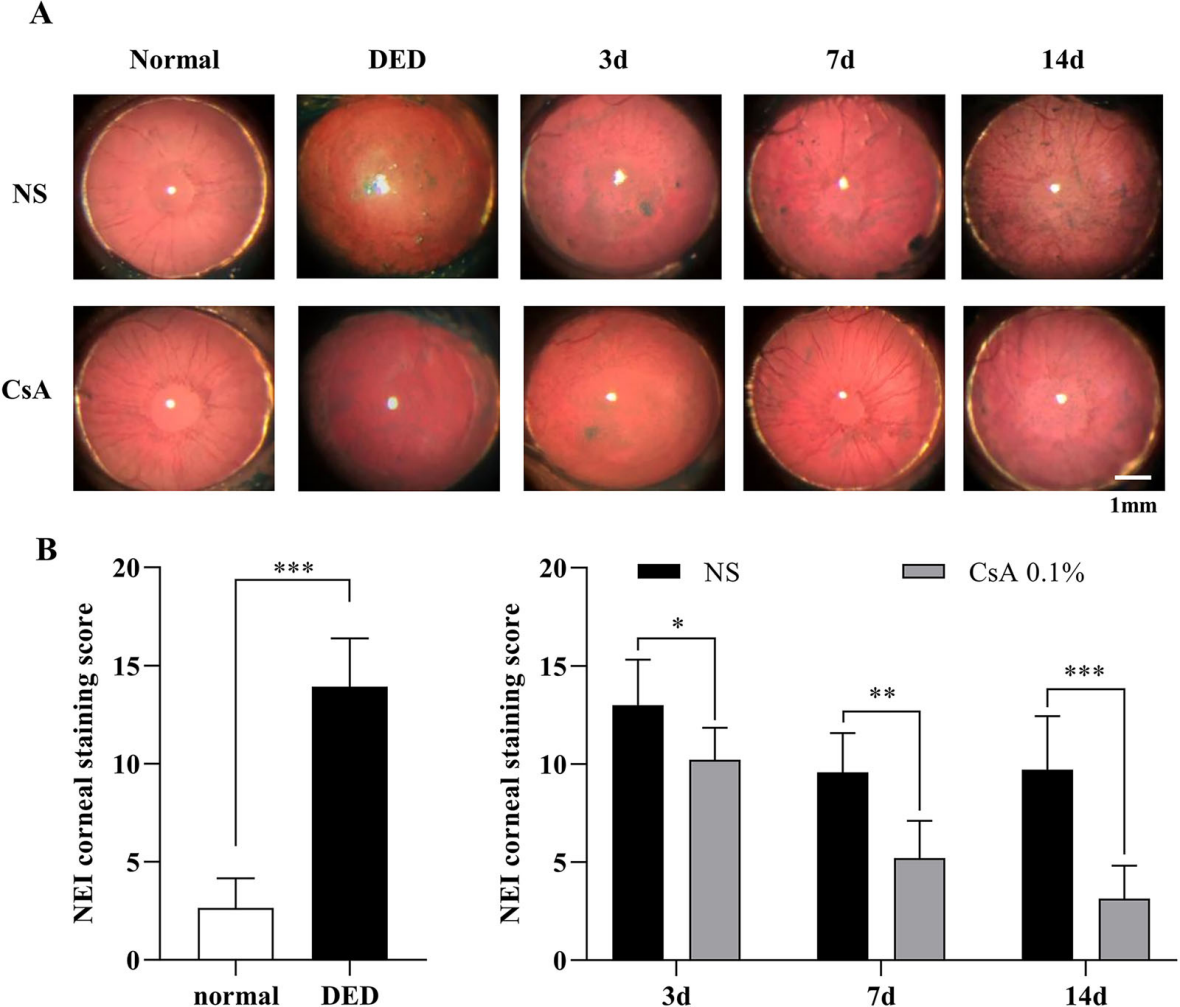
Corneal staining score analysis of DED mice after DED induction and during treatment. (**A**) Corneal staining images with lissamine green in all the experimental groups, including normal mice, NS-treated DED mice, and CsA-treated DED mice, during treatment. (**B**) Corneal staining scores of the normal and DED mice and longitudinal changes in corneal staining scores in the NS-treated and CsA-treated DED groups during treatment. Data are presented as the mean ± standard deviation, and corneal staining scores of the normal and DED mice were compared with the *t* test and Mann–Whitney *U* test. Staining scores of the NS-treated and CsA-treated DED groups on days 3, 7, and 14 of treatment were compared with the multiple *t* test. Statistical significance was assessed using the Holm–Sidak method, *N* = 7, *P < 0.05.

**Figure 6.**
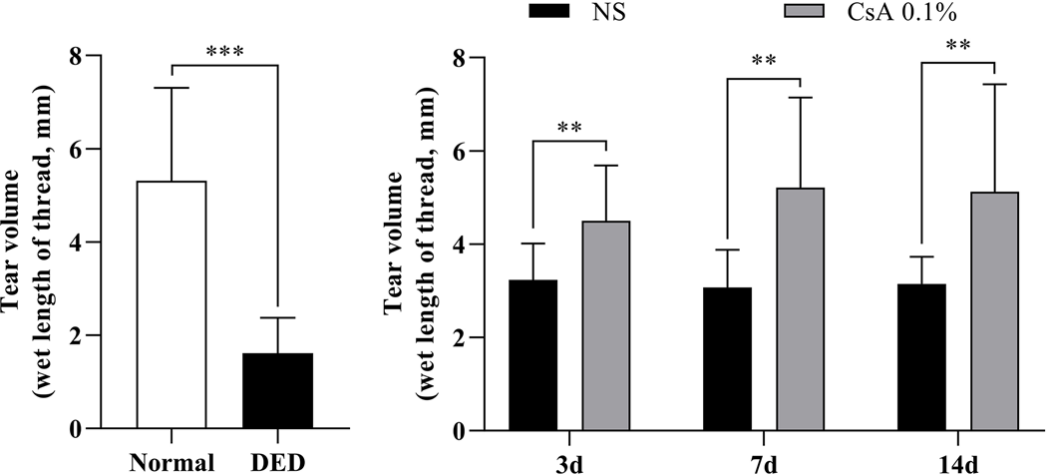
Tear volume analysis of DED mice after DED induction and during treatment. Graphs showing tear lengths of the normal and DED mice and the longitudinal variation in tear length in the NS-treated and CsA-treated DED groups during treatment. Data are presented as the mean ± standard deviation. Tear lengths of the normal and DED mice were compared with the *t* test and Mann–Whitney *U* test. Tear lengths between the 2 treatment groups on days 3, 7, and 14 were compared with the multiple *t* test. Statistical significance was assessed using the Holm–Sidak method, *N* = 7, *P < 0.05.

Various clinical DED evaluation metrics, including corneal irregularity score, corneal staining score, and PRT, were calculated for DED mice after DED induction and during treatment. The evaluation results are summarized in Figures 4–6. The analysis of the corneal irregularity score in DED mice is summarized in Figure 4. The corneal irregularity score of DED mice (4.71 ± 0.49) was significantly higher than that of normal mice (0.86 ± 0.69, *P* < 0.0006). The corneal irregularity scores of the NS-treated DED group remained high until the end of the treatment period (day 3, 4.14 ± 1.07; day 7, 3.43 ± 1.13; and day 14, 3.43 ± 1.62), whereas the scores of the CsA-treated DED group decreased to the level of normal mice during the treatment period (day 3, 2.00 ± 1.00; day 7, 1.00 ± 0.82; and day 14, 0.86 ± 0.38). The irregularity scores of the two treatment groups showed statistically significant differences on all measurement days of treatment (day 3, *P* < 0.002; day 7, *P* < 0.0006; and day 14, *P* < 0.0014; see Fig. 4).

The corneal staining score analysis of DED mice after induction and during treatment is summarized in Figure 5. The corneal staining score with lissamine green was significantly higher in DED mice (13.9 ± 2.5) than in normal mice (2.6 ± 1.5, *P* < 0.0006). During the treatment period, the corneal staining scores remained high in the NS-treated DED group (day 3, 13.00 ± 2.31; day 7, 9.57 ± 2.01; and day 14, 9.71 ± 2.72), whereas the scores in the CsA-treated DED group decreased with time (day 3, 10.21 ± 1.63; day 7, 5.21 ± 1.89; and day 14, 3.14 ± 1.68). The staining scores of the two treatment groups were significantly different on all measurement days (day 3, *P* < 0.022; day 7, *P* < 0.0012; and day 14, *P* < 0.0001).

The results of the PRT for the DED mice are summarized in Figure 6. After DED induction, the amount of tear secretion in the DED group (1.62 ± 0.76) was significantly lower than that in the control group (5.31 ± 1.99, *P* < 0.0001). During treatment, the amount of tear secretion increased slightly in the NS-treated DED group (day 3, 3.23 ± 0.78; day 7, 3.08 ± 0.80; and day 14, 3.14 ± 0.59), but it did not change from day 3 to day 14 of treatment. On the other hand, the amount of tear secretion increased significantly in the CsA-treated DED group on day 3 (4.50 ± 1.19). The amount on days 7 (5.21 ± 1.93) and 14 (5.12 ± 2.30) was close to normal. The difference in tear secretion between the NS-treated and CsA-treated DED groups was significant on all measurement days (day 3, *P* < 0.0054; day 7, *P* < 0.0018; and day 14, *P* < 0.0086).

### Correlation Analysis

**Figure 7.**
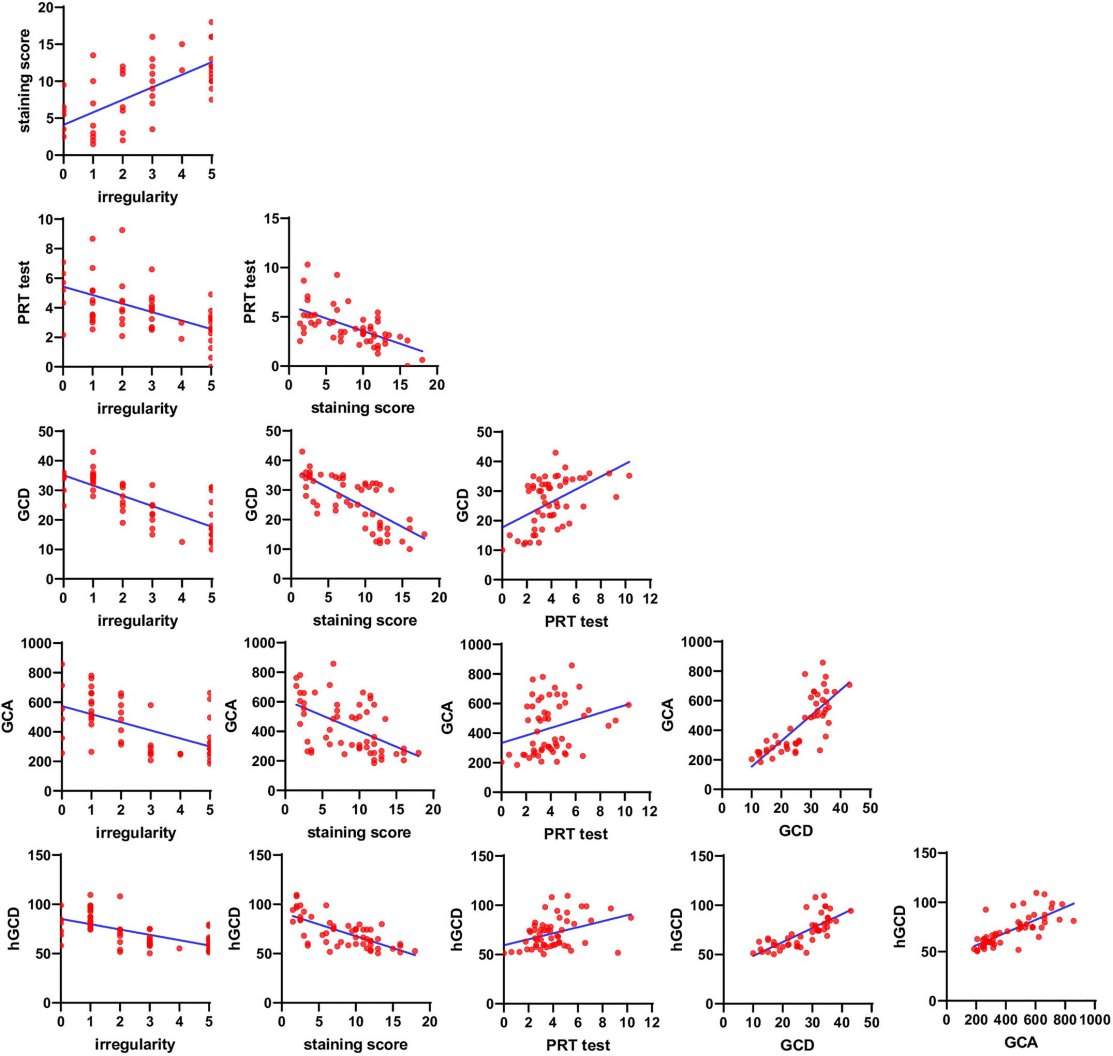
Correlation analysis was conducted using scatterplots for each test, and linear regression analysis was performed to assess the relations. The data showing the correlation between the two groups are presented in Table 2. GCD, goblet cell density; GCA, goblet cell area; PRT, phenol-red thread test.

**Figure 8.**
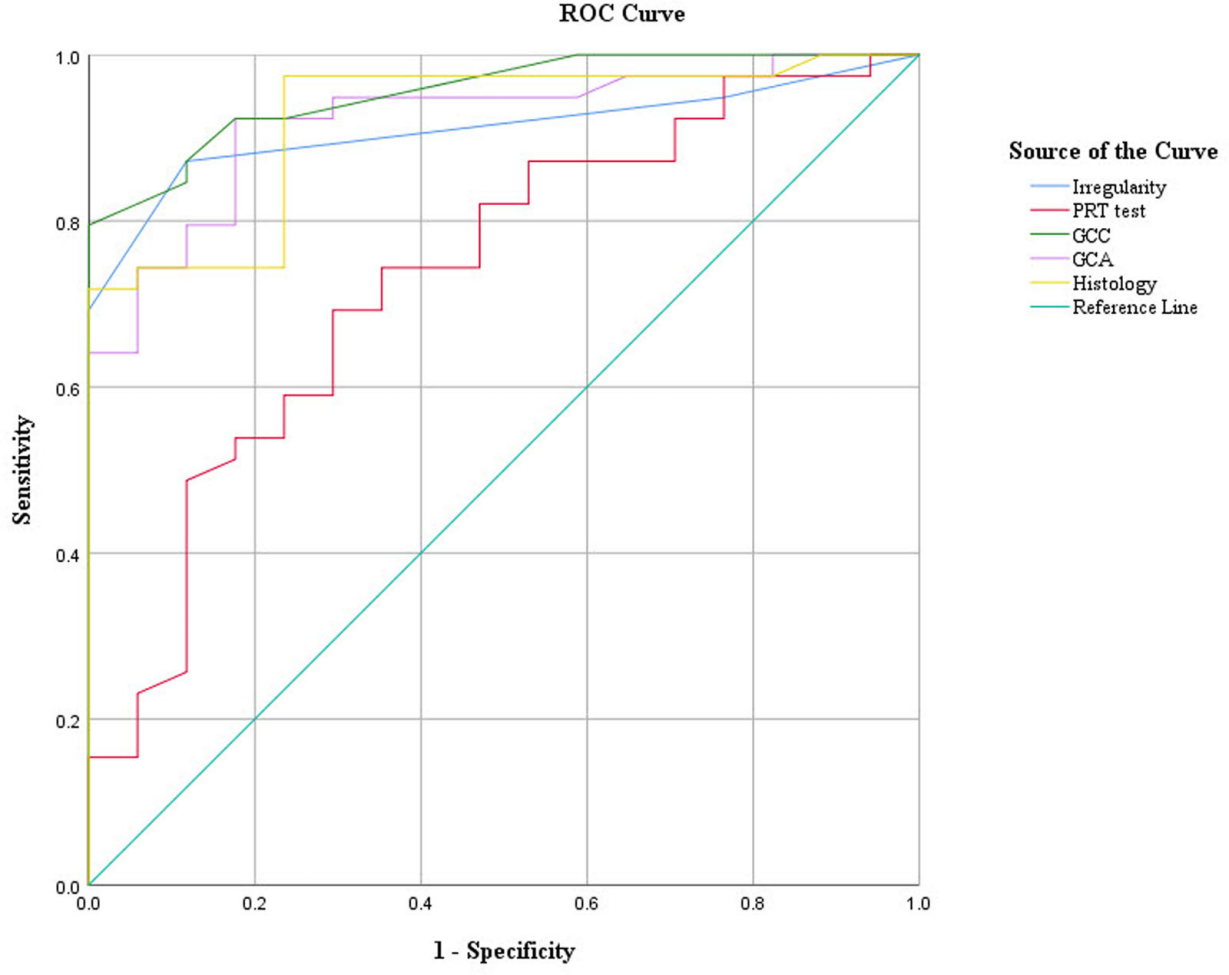
Receiver operating characteristic (ROC) curves of DED parameters, including corneal irregularity, PRT, in vivo goblet cell (GC) parameters, and histology-derived GCD.

Correlation analysis among the GCDs and GCAs from in vivo MBFM imaging, the clinical DED parameters, and the GCDs from PAS histology (hGCD) was performed. Table 1 and Figure 7 show the correlation analysis results of all the data measured. Scatter plots of experimental data pairs, correlation coefficients (*r* values), and significance levels (*P* values) are presented. The GCD and GCA from in vivo MBFM in DED-induced mice showed significant correlations with the corneal irregularity score, corneal staining score, and PRT length. The in vivo GCD and GCA were obviously and closely correlated with ex vivo hGCD. ROC curve analysis was performed to determine the diagnostic values of the various DED parameters. Figure 8 and Table 2 present the ROC curves and the best cutoffs of the variables for achieving optimal sensitivity and specificity. The GCD and GCA from in vivo MBFM showed good predictive ability with high area under the curve (AUC) values.

**Table 1.** Correlation Analysis Among DED Evaluation Metrics, Including Corneal Irregularity Score, Corneal Staining Score, PRT Length, GCD and GCA from In Vivo MBFM, and hGCD From Ex Vivo Histology

	Corneal Irregularity	Corneal Staining Score	PRT Test	GCD (Live Image)	GCA (Live Image)	hGCD (PAS Stain)
Corneal irregularity						
Spearman correlation	1					
Sig. (2-tailed)						
*N*					
Corneal staining score						
Spearman correlation	0.655^**^	1			
Sig. (2-tailed)	<0.001					
*N*	56					
PRT test						
Spearman correlation	−0.537^**^	−0.602^**^	1			
Sig. (2-tailed)	<0.001	<0.001				
N	56	56				
GCD (live image)						
Spearman correlation	−0.753^**^	−0.733^**^	0.532^**^	1		
Sig. (2-tailed)	<0.001	<0.001	<0.001			
*N*	56	56	56			
GCA (live image)						
Spearman correlation	−0.535^**^	−0.582^**^	0.296^*^	0.805^**^	1	
Sig. (2-tailed)	<0.001	<0.001	0.027	<0.001		
*N*	56	56	56	56		
hGCD (PAS stain)						
Spearman correlation	−0.635^**^	−0.688^**^	0.381^**^	0.818^**^	0.789^**^	
Sig. (2-tailed)	<0.001	<0.001	0.004	<0.001	<0.001	
*N*	56	56	56	56	56	1

Spearman correlation analysis was performed.

* Correlation is significant at the 0.05 level (2-tailed).

** Correlation is significant at the 0.01 level (2-tailed).

**Table 2.** Area Under the Curve (AUC) of MBAS-WFFM Parameters With Their Standard Error (SE), 95% Confidence Interval (CI), and Their Best CutOffs for Optimizing Sensitivity and Specificity to Separate Normal and DED Mice

Parameters	Cutoff Point	Sensitivity (%)	Specificity (%)	AUC	SE	95% CI
Irregularity	>1.5	87.18	88.24	0.910	0.039	0.833–0.987
PRT test	>3.87	69.23	70.59	0.735	0.073	0.592–0.877
GCD	<31.5	87.18	88.24	0.955	0.024	0.907–1.002
GCA	<535	82.10	82.35	0.849	0.056	0.739–0.959
hGCD	<73.83	79.49	82.35	0.911	0.038	0.837–0.985

## Discussion

According to the consensus on the multifactorial pathophysiological analysis of DED, there is a lack of correlation between symptoms and clinical signs.^15^ Gold-standard diagnostic examination parameters have not been identified, and the diagnosis and monitoring of DED remain difficult. Various attempts have been made to establish a standard diagnostic method for DED.^16^ The Dry Eye Workshop (DEWS) II of the Tear Film and Ocular Society (TFOS) determined and proposed the most efficacious battery of tests, which starts with the evaluation of the degree of subjective symptoms followed by detailed examinations of the ocular surface and tear film.^17^

Among these tests, the evaluation of ocular surface damage by desiccation stress is essential, and ocular surface staining is extensively used in clinical practice. However, there is no simple and noninvasive evaluation method for assessing GC deficiency in DED, although the damage to conjunctival epithelial cells can be evaluated using conventional staining methods. The evaluation of GC status is crucial in the diagnosis and treatment of DED because the GCD is closely related to the severity of DED and responds sensitively to anti-inflammatory treatment and withdrawal.^18^ Conjunctival impression cytology (CIC) has been established to investigate histological and immunochemical changes in ocular surface diseases as a simple form of conjunctival biopsy.^19^ Although CIC can be used to evaluate the status of GCs in DED patients, CIC has various limitations, including a long processing time of approximately 1 hour, potential discomfort and irritation in some patients, and unavoidable additional ocular surface damage.^20^ Additionally, the CIC protocol is not standardized and cannot be adopted as a routine examination for DED evaluation in clinical practice.^21^^,^^22^ To overcome these limitations, direct noninvasive imaging of GCs has been investigated. Reflectance confocal microscopy (RCM) is based on light reflection, and GCs are visualized as hyper-reflective large round cells relative to other epithelial cells.^23^ Although RCM has potential as a GC examination procedure, it produces images of relatively low contrast and a small imaging FOV of a few hundred micrometers on one side.^24^^,^^25^ We recently reported that MBFM could visualize conjunctival GCs with high contrast via GC-specific labeling with moxifloxacin ophthalmic solution. A noncontact MBFM technique was developed and applied to disease animal models, including chemical burn mouse models and povidone iodine-induced ocular surface damage rabbit models.^6^^,^^26^ In this study, we applied MBFM to the DED mouse model and observed that the in vivo GCD and GCA were highly correlated with clinical evaluation metrics. In addition, ROC curve analysis showed that this system has predictive and diagnostic capabilities as high as those of clinical DED diagnostic methods, including the PRT test, corneal staining, and corneal irregularity score analysis.

In the context of GC imaging in mice, it was observed that the upper eyelids were easier to open and fixate during the process. Notably, previous studies have reported no significant difference in the number of GCs between the lower and upper eyelids within individual mice and variations in GC distribution were more pronounced in the nasal, middle, and temporal regions.^27^ To minimize experimental errors, a pilot test was conducted prior to the main experiment, which confirmed the aforementioned findings (data not shown). In this experiment, comprehensive imaging of the entire GC population on the upper eyelid was conducted, whereas the analysis specifically focused on the GCs in the middle fornix.

The GCD and GCA from in vivo MBFM imaging were highly correlated with the results of histological GCD analysis through PAS staining. The in vivo GCD and GCA showed high accuracies in DED diagnostic ability, although the time required for analysis was considerably short and the cost of examination could be low. MBFM is more positive than CIC in future human clinical trials. Therefore, MBFM will not only have high diagnostic value in the actual clinical environment but can also be used as an important tool for evaluating the severity of DED and its treatment and progression.

This study has several limitations. Although our aim was to establish diagnostic criteria for DED in mice and explore their potential relevance in humans, further research must be required to determine the diagnostic applicability of these findings in humans. Additionally, the imaging equipment used in this study has certain characteristics that may not accurately reflect the density of GCs in the entire conjunctiva but instead capture density information in specific regions, introducing the possibility of error. Therefore, caution should be exercised when interpreting the results, considering the limited coverage of GC density by the imaging device. The current MBFM technique used in this study has an imaging speed of approximately 1 frame per second (fps) and may be susceptible to motion artifacts during in vivo imaging. Future human trials could explore using a new MBFM technique with an imaging speed of more than 10 fps. Such advancements would enhance the reliability and effectiveness of the imaging process.^6^^,^^7^^,^^28^

Furthermore, we treated a 0.5% moxifloxacin ophthalmic solution for in vivo staining, which exhibits intrinsic fluorescence with peak excitation and emission wavelengths at 290 nm and 500 nm, respectively.^5^ To visualize the fluorescence of moxifloxacin, we utilized a 405 nm LED light source. It is important to note that blue light has been associated with the overproduction of reactive oxygen species (ROS) not only in the retina but also in corneal and conjunctival cells.^29^ We did not investigate the generation of ROS in the conjunctiva in response to blue light exposure in this experiment. Thus, when translating these findings into clinical applications, careful consideration and experiments should be made to minimize the amount of blue light exposure and to analyze the effects.

Although additional human clinical trials on DED are necessary, in vivo MBFM can prove useful as an additional battery test alongside other measures, such as tear volume, ocular surface staining score, tear film stability, and tear osmolarity testing.
